# Parvovirus B19-Induced Aplastic Crisis and Hemophagocytic Lymphohistiocytosis in a Child With Hereditary Spherocytosis

**DOI:** 10.7759/cureus.97045

**Published:** 2025-11-17

**Authors:** Mizuki Oyama, Kenichi Sakamoto, Eri Okura, Shoji Saito, Yozo Nakazawa

**Affiliations:** 1 Pediatrics, Shinshu University School of Medicine, Matsumoto, JPN

**Keywords:** anemia, aplastic crisis, hemophagocytic lymphohistiocytosis (hlh), hereditary spherocytosis, human parvovirus b19 (b19v)

## Abstract

Parvovirus B19 (PVB19) is a common virus that usually causes a mild and self-limiting illness. However, in patients with hereditary spherocytosis (HS), it can lead to severe complications such as aplastic crisis and, rarely, hemophagocytic lymphohistiocytosis (HLH). We report a six-year-old boy with HS who presented with persistent fever and severe anemia. Laboratory tests revealed a progressive pancytopenia, including a sudden drop in hemoglobin from 10 to 6.8 g/dL, hyperferritinemia (5,129 ng/mL), elevated soluble IL-2 receptor (1,179 U/mL), mild hypofibrinogenemia, and positivity for PVB19 IgM. Bone marrow examination revealed hypercellularity with the absence of mature erythroblasts, numerous giant proerythroblasts, and hemophagocytic histiocytes, findings consistent with PVB19-associated aplastic crisis and HLH. The patient was treated with intravenous immunoglobulin and prednisolone, resulting in rapid resolution of fever and hematologic recovery. PVB19-induced HLH is extremely rare, accounting for a small percentage of infection-associated HLH cases in Japan. This case underscores the need for vigilance regarding PVB19 infection in HS patients, as overlapping aplastic crisis and HLH may cause rapid anemia progression and organ dysfunction if not promptly recognized and treated.

## Introduction

Parvovirus B19 (PVB19) is a common childhood virus that typically causes erythema infectiosum, characterized by a “slapped cheek” facial rash. However, it is usually self-limiting in healthy children. PVB19 can cause various complications, including arthritis, myocarditis, meningitis, and hematologic disorders, such as hemophagocytic lymphohistiocytosis (HLH) [1,2]. Hereditary spherocytosis (HS) is a congenital disorder of the red blood cell membrane caused by defects in proteins such as spectrin, ankyrin, and band 3. This leads to spherical red blood cells, hemolysis, and splenomegaly [3]. Patients may experience an aplastic crisis triggered by a PVB19 infection. This results in a sudden drop in hemoglobin due to transient bone marrow suppression. As PVB19 can cause an aplastic crisis in patients with underlying HS, special attention should be paid to the complications owing to PVB19 infections in patients with HS. HLH is a hyperinflammatory syndrome characterized by uncontrolled activation of macrophages and cytotoxic T cells, resulting in excessive cytokine release and multiorgan dysfunction. HLH can sometimes progress to a severe, life-threatening condition [4]. Here, we report a case of PVB19 infection complicated by both aplastic crisis and HLH in a child with HS.

## Case presentation

**Figure 1:**
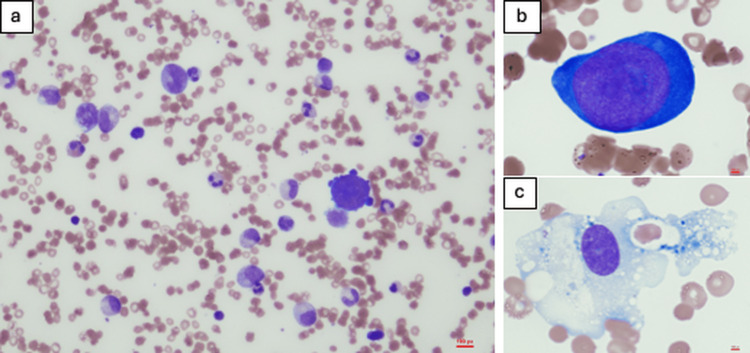
Bone marrow aspiration of the patient. (a) Hypercellular bone marrow with absence of mature erythroblasts. (b) Giant proerythroblast. (c) Hemophagocytic histiocyte.

A six-year-old boy with underlying HS was referred to a local hospital for fever evaluation. Blood examination revealed a sudden drop in hemoglobin (Hb) levels from 10 to 6.8 g/dL, and he was referred to our hospital because of a suspected aplastic crisis. After being admitted, he received a red blood cell transfusion and was evaluated for pyrexia. Additional blood tests at our hospital revealed mild leukopenia (WBC: 3,870 /μL) and platelets (PLT: 24.7 × 10^4^/μL), with hyperferritinemia (5,129 ng/mL), elevated soluble IL-2 receptor levels (1,179 U/mL), a mild decrease in fibrinogen (227 mg/dL), and positivity for PVB19 IgM (12.7 by enzyme immunoassay). The key laboratory findings at admission to our hospital are shown in Table 1. On the third day of hospitalization, the fever persisted, and pancytopenia progressed (WBC: 1,760 /μL, Hb: 8.4 g/dL (after red blood cell transfusion), and PLT: 11.4 × 104/μL). We suspected not only aplastic anemia but also the presence of HLH complications, so we performed a bone marrow examination. Bone marrow aspiration revealed a hypercellular bone marrow (nuclear cell counts: 30,200 /μL), an absence of mature erythroblasts (Figure 1A), and giant proerythroblasts (Figure 1B), which were characteristic findings of aplastic crisis due to PVB19 infection [5]. Additionally, hemophagocytic histiocytes were observed in the bone marrow (Figure 1C). Based on the persistent fever, pancytopenia, hypofibrinogenemia, hyperferritinemia, elevated soluble IL-2 receptor levels, and the presence of hemophagocytic features in the bone marrow, we diagnosed HLH based on the HLH-2004 clinical trial diagnostic criteria (Table 2) [6]. Thus, the patient was diagnosed with aplastic crisis and HLH due to acute PVB19 infection. After the administration of intravenous immunoglobulin and prednisolone for HLH, the patient's fever resolved quickly, and hematological recovery was achieved. The patient was discharged on the seventh day of hospitalization. After being discharged, the patient developed a rash, a common symptom of PVB19. However, they have been doing well since then, without signs of anemia or a recurrence of HLH.

**Table 1:** Key laboratory findings at the admission to our hospital.

Laboratory findings	Results	Reference range
White blood cells (/μL)	3,870	3,800 - 10,100
Hemoglobin (g/dL)	6.5	11.9 - 14.9
Platelet count (/μL)	247,000	180,000 - 440,000
Aspartate aminotransferase (U/L)	49	13 - 33
Alanine aminotransferase (U/L)	15	7 -23
Total bilirubin (mg/dL)	2.77	0.4 - 1.5
Alkaline phosphatase (U/L)	133	105 - 483
Lactate dehydrogenase (U/L)	460	145 - 270
Ferritin (ng/mL)	5,129	25 - 280
Soluble IL-2 receptor (U/mL)	1,179	155 - 475
Fibrinogen (mg/dL)	227	180 - 350
Parvovirus B19 IgM	12.7	Negative

**Table 2:** Diagnostic criteria for hemophagocytic lymphohistiocytosis fulfilled in our case during admission to our hospital.

Diagnostic criteria	Fulfilled in our case (Yes/No)
1. Fever	Yes
2. Splenomegaly	No
3. Cytopenias (affecting ≧ 2 of 3 lineages in the peripheral blood:	Yes
Hemoglobin (<9.0 g/dL); Platelets (<100 x 10^9^/L); Neutrophils (<1.0 x 10^9^/L)	
4. Hypertriglyceridemia and/or hypofibrinogenemia	Yes
Fasting triglycerides ≧ 3.0 mmol/L; Fibrinogen ≦ 150 mg/dL	
5. Hemophagocytosis	Yes
6. Low or absent natural killer (NK) cell activity (according to local laboratory reference)	Not evaluated
7. Ferritin ≧ 500 ng/mL	Yes
8. Soluble IL-2 receptor ≧ 2,400 U/mL	No

## Discussion

Our patient had underlying HS and presented with pancytopenia, including rapidly progressive anemia due to an aplastic crisis, as well as HLH caused by PVB19 infection. PVB19 infection typically presents asymptomatic or with only mild symptoms in healthy individuals, but it can occasionally lead to serious complications such as HLH, myocarditis, and arthritis. Furthermore, patients with HS as an underlying disease should be cautioned as they may develop aplastic crisis. Aplastic crisis occurs when PVB19 infects erythroid progenitor cells in the bone marrow, leading to transient suppression of erythropoiesis [1]. In patients with HS or other hemolytic anemias, this temporary cessation of red cell production can cause a sudden and severe drop in hemoglobin levels. In addition, HLH is a hyperinflammatory syndrome resulting in excessive cytokine release and multiorgan dysfunction. Previous clinical trial for patients with HLH revealed that five-year overall survival was 61% (95% CI: 56-67%) [7]. Early recognition and prompt initiation of diagnostic evaluation and treatment are essential in HLH, as delayed management can lead to rapid clinical deterioration and poor outcomes. HLH is an inflammatory disease that is divided into two subtypes: primary HLH and secondary HLH. Primary HLH is a genetic disorder, while secondary HLH is caused by various triggers, such as infection, malignancy, autoimmune disease, and drugs [4]. Infectious diseases are the most common cause of secondary HLH in Japan. Among the 301 patients with infection-associated HLH, Epstein-Barr virus was the most common cause of HLH (163/301, 54%), and PVB19-induced HLH was reported in 3/301 (1%) [8]. Previously, two cases of HLH and aplastic crisis complicated by PVB19 were reported in patients with underlying HS [5,9]. Both cases presented pancytopenia, especially severe anemia (Hb: 3.9 g/dL and 5.6 g/dL), and required red blood cell transfusions. The details of these two cases are presented in Table 3. HLH is a life-threatening disease that can cause multiple organ failure owing to an excessive immune response. The relationship between HLH and aplastic crisis remains unclear, but both conditions cause anemia. An aplastic crisis inhibits erythrocyte production, while HLH destroys erythrocytes. Erythrocyte production recovers during recovery from aplastic crisis; however, if HLH coexists, anemia persists due to erythrocyte destruction. In other words, HLH worsens anemia caused by aplastic crisis. Since pancytopenia is a primary symptom of HLH, patients with HS who have already developed an aplastic crisis due to PVB19 infection have an increased risk of rapid progression to anemia. At the onset of HLH, there is often a fever. Due to the increased oxygen demand, it is important to be cautious because the risk of multiple organ failure increases when vital organs do not receive enough oxygen due to the rapid progression of anemia.

**Table 3:** Previous case reports of HLH and aplastic crisis complicated by PVB19 with underling HS. HLH: hemophagocytic lymphohistiocytosis; PVB19: parvovirus B19; HS: hereditary spherocytosis; Hb: hemoglobin; F: female; PLT: platelets; sIL2-R: soluble IL-2 receptor; N.D.: not determined; WBC: white blood cells.

Case	Age	Sex	WBC (×10^9^/μL)	Hb (g/dL)	PLT (×/μL)	Ferritin (ng/mL)	sIL2-R (U/mL)	Fibrinogen (g/L)	Bone marrow examination	Treatment	Outcome	Reference
1	17	F	1,900	5.6	74,000	7,428	N.D.	2.31	Hypocellular bone marrow, giant proerythroblast, absence of mature erythroblasts, hemophagocytic histiocyte	Red blood cell transfusion, intravenous immunoglobulin	Alive	Yilmaz et al. [5]
2	7	F	2,840	3.9	41,000	18,525	2,483	2.16	Hypercellular bone marrow, giant pro-erythroblast, erythroid hypoplasia, hemophagocytic histiocyte	Red blood cell transfusion, chemo-immunotherapy (etoposide, dexamethasone, and cyclosporin A)	Alive	Kim et al. [9]
This Case	6	M	1,760	8.4	114,000	5,129	1,179	2.27	Hypercellular bone marrow, giant pro-erythroblast, erythroid hypoplasia, hemophagocytic histiocyte	Red blood cell transfusion, intravenous immunoglobulin, prednisolone	Alive	-

## Conclusions

In patients with HS, the simultaneous occurrence of aplastic crisis and HLH causes severe bone marrow suppression and excessive immune activation, leading to synergistically rapid progression of anemia and multiple organ failure. Early recognition of this rare but critical overlap by clinicians is crucial to initiate appropriate management and prevent fatal outcomes. This case highlights the need for special caution regarding parvoviral infection in patients with underlying diseases, and proper diagnosis and treatment are necessary.
